# Crime

**Published:** 1874-07

**Authors:** 


### CRIME.

DISEASE and premature death are the usual concomitants the world over. The greatest wisdom is to prevent disease, and it is practicable. On the other hand, crime can also be prevented to a very great extent. It is very clear that education simply makes no strikingly perceptible decrease either in the frequency or in the enormity of crimes. Millions of money are spent in maintaining public schools in the State of New York, but the three State prisons, and the sixty-seven jails, and the five juvenile reformatories, and the six penitentiaries under State control, are more full of occupants to-day than at any previous time of our history. These three State prisons, with their three thousand occupants, cost more annually than the two hundred and ten academies in the State, with their thirty thousand students; and yet, with all this, there are multitudes of men—many of them educated and occupying high positions in society—who are clamoring for taking the Bible out of the public schools, so that it shall not even be read; claiming, furthermore, many of them, that there shall be no religious instruction imparted, nor even the repetition of a prayer, and for this, among other reasons, that there ought to be no connection between Church and State, and that the minds of the young should not be biassed in matters of religion. The trouble really is in the fact that there is too little religious teaching and reading in the schools where our children are instructed; their education should be more Christian than it is; and it may become a question with religious parents whether the best interests of their children may not require that they be withdrawn from the public schools, where so little religion is taught, and placed under instructions which necessarily involve more religious training, in the form of denominational schools. A new element of crime within a few years past has sprung up, not regarded by any dozen persons in a hundred, and, unless counteracted in some way, will add largely to the victims of the penitentiary and the gallows, confined as yet, however, to cities and large towns. It is found in what is called "Labor Organizations." There are men in New York who are called "contractors," or "builders," and who, by their enterprise, have done more than any class of men to enlarge, and beautify, and enrich our city. If you want a house built, or if Mr. Astor or Mr. A. T. Stewart desire to erect a dozen or more private dwellings or stores, a contractor is ready to undertake it for a certain number of dollars, agreeing to furnish all the materials, and employ all the masons, carpenters, plasterers and others needed, and he agrees to deliver the keys on a certain day. One of these energetic men undertakes a million dollars' worth of buildings in a single summer, but, when they are half done, his workmen come to him some sunny morning and say, "We will quit work to-night unless you dismiss that apprentice boy whom you have sent to work with us to-day; you have four apprentices already, if you keep on taking them they will soon be able to do the work as well as we can, and you will dismiss us, because they cost you but a dollar a day and you give us four." The contractor has to yield, because he is under obligation to have the house completed by a certain day, and if not, he forfeits ten, fifty or a hundred or more dollars a day until the work is done. He has not time to engage new workmen, and, even if he had, these new men would not be allowed to work—they would be waylaid and beaten, if not killed, by those workmen whom the contractor has under his present employ. These hundred men may average two sons apiece, and out of these two hundred only four can learn the trade; they cannot go to other trades, because similar rules hold; then it follows that a hundred and ninety-six boys out of two hundred are prevented from learning any regular trade which requires intelligence and skill, with the result that they hang around street corners, and take up with what odd jobs they can get of any kind of menial or days' work. From bad weather or other causes there is nothing to do for many days in the year, but they must eat, and the inevitable result is that they learn to gamble, and cheat, and steal in multitudes of cases. Some of these two hundred boys may go to the country or emigrate westward, and others may get an education fitting them for higher positions, but two thirds of them will grow up idle and shiftless, and life comes to a premature if not criminal ending. Until some better thing is proposed to meet so serious a condition of affairs, means might be adopted through government, general and State, to take these youths and educate them as soldiers for the army, and as sailors for the navy and mercantile vessels. The man or woman who shall mature this plan and make it easily practicable, will deserve well of his country, for it would save many a youth from an aimless life, from idleness, beggary and crime.

